# To Increase Weight

**Published:** 1887-06

**Authors:** 


TO INCREASE WEIGHT.—Eat to the extent of satisfying a natural appetite, of fat meats, butter, cream, milk, cocoa, chocolate, bread, potatoes, peas, parsnips, carrots, beets, farinaceous foods, as Indian corn, rice, tapioca, sago, corn-starch pastry, custards, oatmeal, sugar, sweet wines, and ale. Avoid acids; exrercise as little as possible; sleep all you can, and don't worry or fret.—*Ibid.*

